# Diagnostic implications of ubiquitination-related gene signatures in Alzheimer's disease

**DOI:** 10.1038/s41598-024-61363-1

**Published:** 2024-05-10

**Authors:** Fei Xu, Wei Gao, Miao Zhang, Fuyue Zhang, XiaoFei Sun, Bao Wu, Yali Liu, Xue Li, Honglin Li

**Affiliations:** 1 Heilongjiang Provincial Administration of Traditional Chinese Medicine, Harbin, 150036 Heilongjiang China; 2 Jiangsu College of Nursing, Huaian, 223003 Jiangsu China; 3 Heilongjiang University of Chinese Medicine, Harbin, 150040 Heilongjiang China; 4 Fujian University of Traditional Chinese Medicine, Fuzhou, 350122 Fujiang China; 5 Shanghai University of Sport, Shanghai, 200438 China

**Keywords:** Alzheimer's disease, Ubiquitination, Risk gene, Diagnostic model, CeRNA regulatory network, Neurological disorders, Nutrition disorders, Biochemistry, Computational biology and bioinformatics

## Abstract

The purpose of this study was to explore the diagnostic implications of ubiquitination-related gene signatures in Alzheimer's disease. In this study, we first collected 161 samples from the GEO database (including 87 in the AD group and 74 in the normal group). Subsequently, through differential expression analysis and the iUUCD 2.0 database, we obtained 3450 Differentially Expressed Genes (DEGs) and 806 Ubiquitin-related genes (UbRGs). After taking the intersection, we obtained 128 UbR-DEGs. Secondly, by conducting GO and KEGG enrichment analysis on these 128 UbR-DEGs, we identified the main molecular functions and biological pathways related to AD. Furthermore, through the utilization of GSEA analysis, we have gained insight into the enrichment of functions and pathways within both the AD and normal groups. Further, using lasso regression analysis and cross-validation techniques, we identified 22 characteristic genes associated with AD. Subsequently, we constructed a logistic regression model and optimized it, resulting in the identification of 6 RUbR-DEGs: *KLHL21, WDR82, DTX3L, UBTD2, CISH,* and *ATXN*3L. In addition, the ROC result showed that the diagnostic model we built has excellent accuracy and reliability in identifying AD patients. Finally, we constructed a lncRNA-miRNA-mRNA (competing endogenous RNA, ceRNA) regulatory network for AD based on six RUbR-DEGs, further elucidating the interaction between UbRGs and lncRNA, miRNA. In conclusion, our findings will contribute to further understanding of the molecular pathogenesis of AD and provide a new perspective for AD risk prediction, early diagnosis and targeted therapy in the population.

## Introduction

Alzheimer's disease (AD) is a type of dementia which is characterized by continuous cognitive dysfunction and behavioral defects that occur in old age and the presenile period^1^. The clinical signs are visuospatial impairment, apraxia, aphasia, agnosia, memory impairment, abstract thinking and computing impairment, and personality and behavior change^2^. The occurrence of AD constitutes about 50% to 70% of dementia in old age^3^. The association of environmental and lifestyle, genetic factors is caused in part by particular genetic alterations that result in AD^4^. Currently, AD-treated drugs are not available. In clinical practice, combined drug therapy, careful nursing and non-drug therapy are often used to decrease symptoms and slow down the disease development^5^.

Ubiquitination is a cellular process wherein a low molecular weight protein, ubiquitin mediates the protein classification within the cell. This process involves a sequence of specialized enzymes that choose targeted protein molecules and facilitate specific modifications. The enzymes involved in ubiquitination such as ubiquitin-stimulating enzymes, degrading enzymes, and binding enzymes^6^. Ubiquitination takes place a crucial part in various aspects of protein biology, such as metabolism, localization, function, degradation and regulation. However, it also participates in the control of cell cycle, apoptosis, multiplication, transfer, differentiation, transcriptional regulation, gene expression, damage repair, transmission of signal, inflammation, immunity and nearly all vital life activities^7,8^. Furthermore, ubiquitination exhibits a strong association with the occurrence of tumor and cardiovascular diseases^9,10^. Consequently, owing to its significant contributions to the field of biochemical research, ubiquitination has emerged as a novel focal point for the investigation and advancement of novel pharmaceuticals.

Many neurodegenerative diseases like AD, are a collection of harmful and associated-prone proteins. These associated proteins are found to be ubiquitinated in many neurodegenerative diseases. Even though hazardous proteins are instantly deteriorated by proteolytic systems in healthy individuals, any systems perturbation caused by genetic differences, lifestyle or aging leads to a collection of hazardous protein composites and the outbreak of different diseases like neurodegenerative diseases. The significant targeting signal for proteolytic systems is a Ubiquitination. Ubiquitin-conjugating enzyme E2I (Ubc9) ligates small ubiquitin-related modifier (SUMO) to target proteins, resulting in changes of their localization, activity, or stability. Sumoylation of amyloid precursor protein (APP) was reported to be associated with decreased levels of beta amyloid (Abeta) aggregates, suggesting that sumoylation may play a role in the pathogenesis of AD. The association between genetic variations of Ubc9 gene (UBE2I) and late-onset Alzheimer's disease. Sporadic Alzheimer's disease (SAD) is the leading neurodegenerative disease. With the evolution of next-generation DNA sequencing technology, various inherent risk factors have been illustrated. Studies have shown that more single nucleotide variants (SNVs) have been found in genes that exist on the X chromosome from SAD patients. These variations were validated using the strictest method through the Chain termination method. In loci associated ubiquitin pathway (ATXN3L and UBE2NL) two of the inherent variants were established and have not already been elucidated as SAD inherent risk factors. However, the pathogenesis of AD is not fully understood^11^. Therefore, we speculated that ubiquitination-related genes (UbRGs) have certain diagnostic significance for AD and are involved in controlling the incidence and growth of AD. In this study, we validated our hypothesis through comprehensive bioinformatics analysis to further understand the molecular mechanism of AD.

## Materials and methods

### Sample data acquisition and collation

The Gene Expression Omnibus (GEO) database, established and managed by the National Center for Biotechnology Information (NCBI), encompasses a diverse range of gene expression data, including second-generation sequencing data, chip sequencing data, single-cell sequencing data, and more^12^. We selected and downloaded dataset GSE5281 from the GEO database, which included 87 AD samples and 74 normal samples. DEGs are processed using the “R” language “limma” package and calculate adjusted P values and | logFC|. For GSE5281 gene expression profiles, *P* values < 0.05, and |log (FC)|  > 1were selected as the cutoff values^13^.

### Differential gene expression analysis between AD and normal groups

First, we input the transcriptome expression matrix and the grouping list of samples into R software. If a gene had multiple rows of expression values, its average value was taken, and the expression value was log2-transformed. Next, we cited R package limma to calculate the gene expression difference between the AD group and normal group through the function Wilcox and obtained the differentially expressed genes (DEGs)^14^. Finally, the results of differential expression analysis were output and visualized via R packages pheatmap and ggplot2. The screening conditions were |log2 (fold change, FC)|  > 0.585 and adjusted P-value < 0.05^15^.

### Screening of ubiquitination-related DEGs (UbR-DEGs)

Through the above differential expression analysis, we obtained DEGs between groups. Then we read the result file of the difference analysis and the list of ubiquitination-related genes (UbRGs) from the database iUUCD 2.0 (http://iuucd.biocuckoo.org/)^16^ and took the intersection between DEGs and UbRGs to obtain UbR-DEGs. Furthermore, we utilized the R package VennDiagram to generate a Venn diagram to visualize the UbR-DEGs^17^. Additionally, we employed the R packages limma and pheatmap to visualize the expression patterns of these UbR-DEGs^15^. Fisher's exact test is used to analyze the common genes between DEGs and UbRGs. To exclude non accidental occurrences, paired t-tests were used. When *P* < 0.05, it is considered statistically significant.

### Gene Ontology (GO) and Kyoto Encyclopedia of Genes and Genomes (KEGG) enrichment analyses of UbR-DEGs

The initial step involved the conversion of UbR-DEGs names to R-recognized gene IDs using the R package org.Hs.eg.db^18^. Subsequently, we conducted GO enrichment analysis to explore UbR-DEGs and AD-related genes, including 27 biological processes (BP), cellular components (CC), and molecular functions (MF). This analysis was performed using the R packages cluster Profiler and enrichplot. Following this, the enrich KEGG function was employed to identify the key pathways associated with the molecular mechanisms of AD by enriching the pathways of UbR-DEGs. The outcomes were then visualized using the R packages enrichplot and ggplot2^19,20^. A significance level of P-value < 0.05 was adopted for statistical significance.

### Gene set enrichment analysis (GSEA) between AD and normal groups

GSEA is used to evaluate the distribution trend of genes from a predefined gene set in the gene list ranked with phenotypic relevance, to judge its contribution to phenotype^21^. We used the R packages limma, org. Hs.eg.db, cluster Profiler, and enrichplot to first read and collate the expression parameter information of all transcriptome genes and the customized gene set file. We then performed GSEA enrichment analyses (including functional and pathway enrichment analyses for AD and normal groups). Finally, we visualized the results by enrichment plots^22,23^. A P-value less than 0.05 was considered as significant.

### Identification of hub genes

First, we read the list file of UbR-DEGs, extracted the expression levels of UbR-DEGs according to the transcriptome gene expression matrix, and obtained the grouping information of samples. The “glmnet” package from R software (version 3.6.3) was used to perform the LASSO Cox regression model analysis. The “lambda.1se” value, which was determined by tenfold cross-validation, was set as the lambda for model fitting^24^.

### Logistic regression model

We divided the GSE5281 dataset into a training set and an independent testing set to construct a logistic regression model. The samples were classified into 2 types, the control group and the AD sample group. DEG expression was included as continuous predictive variables, and the sample type was regarded as categorical responsive values to establish the logistic regression model by using glm package in R software. The screened variables by the stepwise regression method were adopted to reconstruct the model, and the p value of each predictive variable was calculated. The variables with *p* value ≤ 0.05 were chosen for model reconstruction and subsequent analysis^25,26^. Receiver operating characteristic (ROC) curves for 1, 3, and 5 years were plotted using area under the curve (AUC) calculated. Finally, the validation set was utilized to assess predictive accuracy and performance of UbR-DEGs the model.

### Construction of lncRNA-miRNA-mRNA (ceRNA) regulatory network based on RUbR-DEGs

To determine the interaction between lncRNAs and RUbR-DEGs (mRNAs), we combined lncRNAs and mRNAs data with miRNAs data respectively to construct lncRNA-miRNA-mRNA (ceRNA) regulatory networks. First, we used TargetScan, miRanda and miRDB databases to predict the target genes (RUbR-DEGs) of miRNAs. If RUbR-DEGs were considered to be the target gene of a specified miRNA in the three databases at the same time, we would retain the RUbR-DEGs for subsequent analysis^27,28^. Then, we used the Perl script to determine the interaction between miRNA and lncRNA through the database spongeScan and obtained the network relationship file and node attribute file^29^. Finally, we used the software Cytoscape to visualize the interaction between lncRNA-miRNA-mRNA and construct the ceRNA regulatory network^30^.
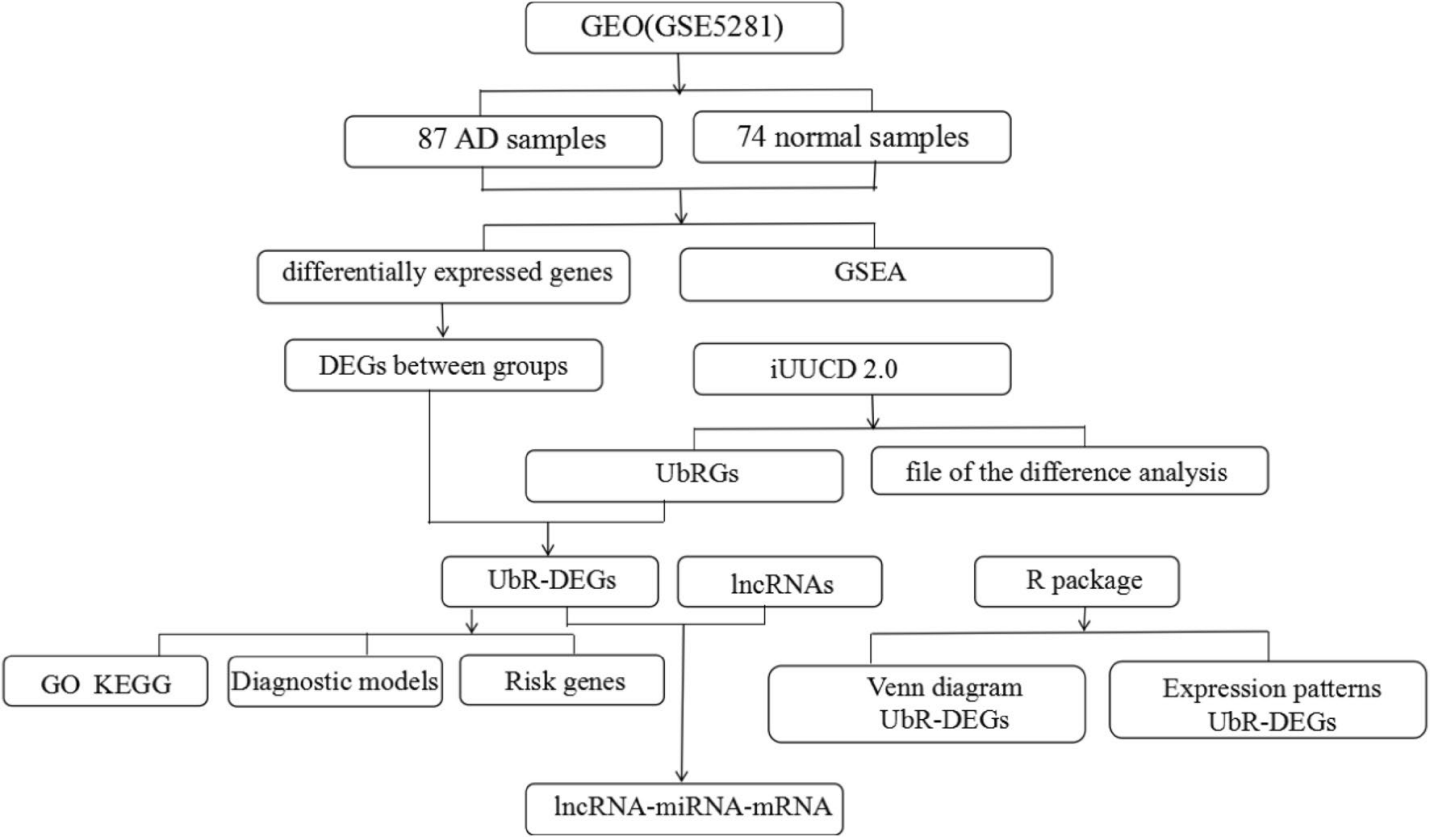


## Results

### GSE5281 dataset clinical characteristics

Transcriptomics data from previously published literature curated in Gene Expression Omnibus (GEO) database was searched for Alzheimer’s disease and only those GEO datasets were selected where the differential gene expression analysis could be done by the GEO2R tool for gene expression data. We have analysed the gene expression datasets with GSE IDs—GSE5281. Represent the pooled data sets from the brain regions: Entorhinal cortex, hippocampus, medial temporal gyrus, posterior cingulate, superior frontal gyrus, and Primary visual cortex. Detailed information of age, number, and gender of subjects is provided in Table 1.

**Table 1:** GSE5281 dataset.

Total number of samples	Sex		Age	
	Male	Female	Male	Female
AD samples (87)	50	37	68–97 years	70–95 years
Normal samples (74)	53	21	63–85 years	73–102 years

### Identification of DEGs between AD and normal groups

**Figure 1:**
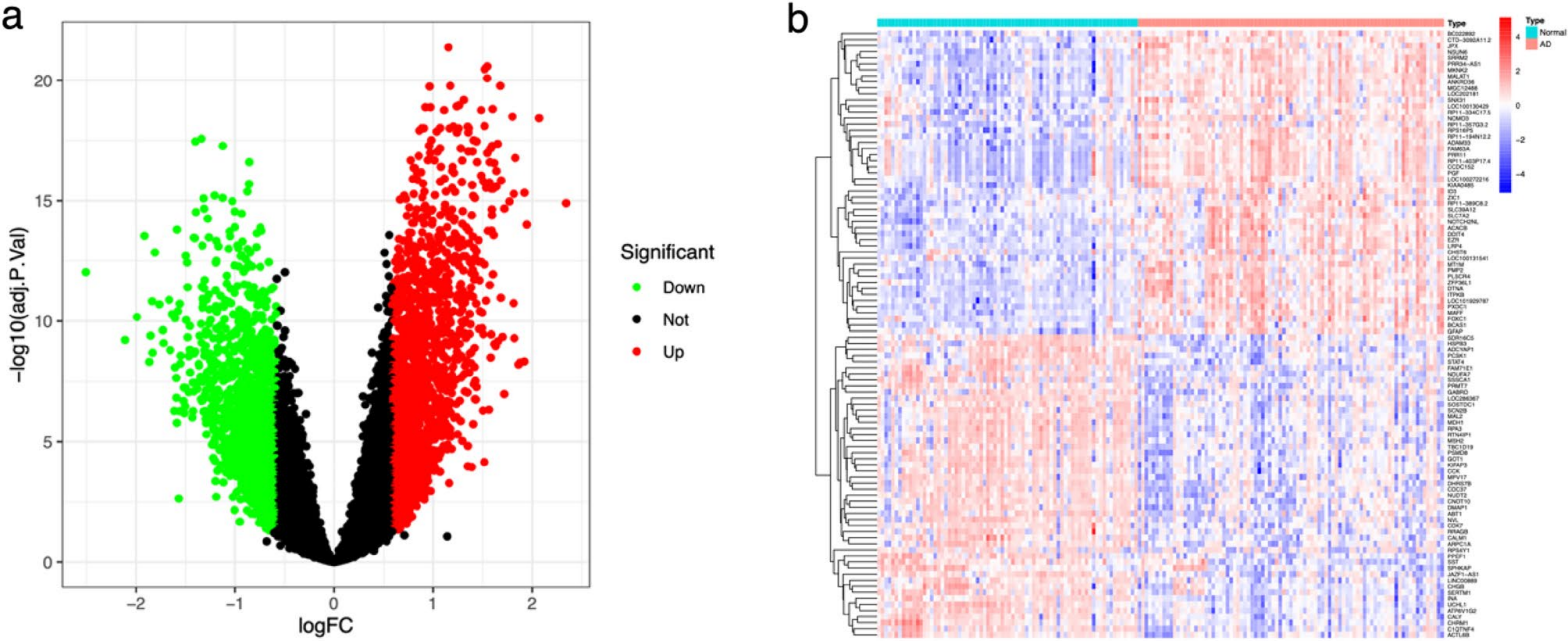
The volcano plot (**a**) and heatmap (**b**) of DEGs expressed between the AD and normal groups. Red dots or squares indicate upregulated DEGs; Green dots or blue squares indicate downregulated DEGs.

By conducting differential expression analysis, a total of 3450 DEGs were identified between the AD and normal groups. Among these DEGs, 1733 were up-regulated and 1717 were down-regulated, as depicted in Fig. 1a. Furthermore, Fig. 1b displayed a heat map illustrating the top 50 up-regulated and down-regulated DEGs.

### Identification of UbR-DEGs in AD

**Figure 2:**
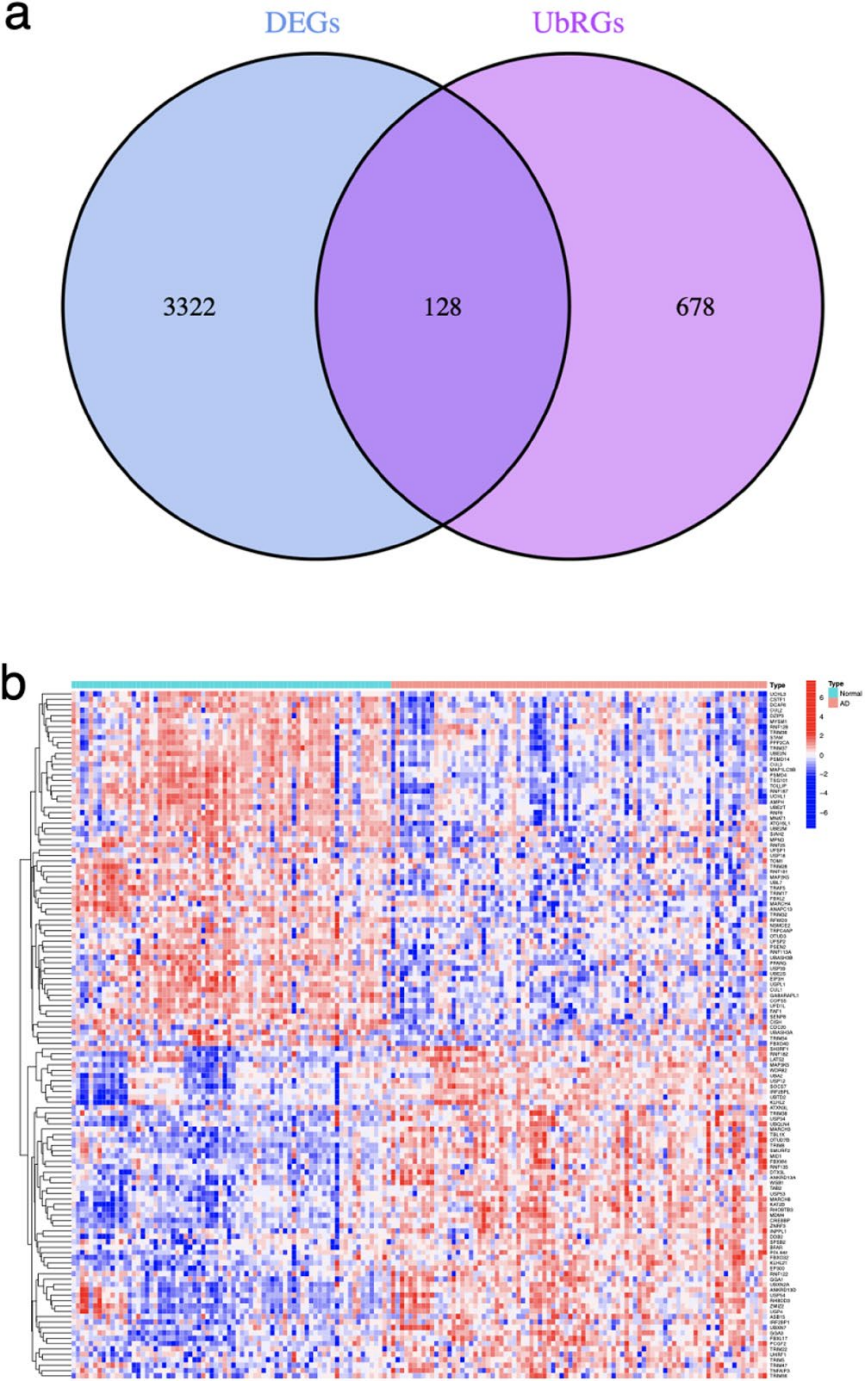
Identification of UbR-DEGs in AD. (**a**) The Venn diagram of overlapped genes (UbR-DEGs). (**b**) The heatmap of UbR-DEGs expressed between the AD and normal groups. Red squares indicate upregulated genes and blue squares indicate downregulated genes.

A total of 3450 DEGs were identified through differential expression analysis, while 806 UbRGs were obtained from the iUUCD 2.0 database. Subsequently, 128 genes were identified as the overlapped genes, referred to as UbR-DEGs (Fig. 2a). The number of UbR-DEGs upregulated is 73, and the number of downregulated is 55.The differential expression of these UbR-DEGs between the AD and normal groups is depicted in Fig. 2b.

### GO and KEGG enrichment of 128 UbR-DEGs in AD

**Figure 3:**
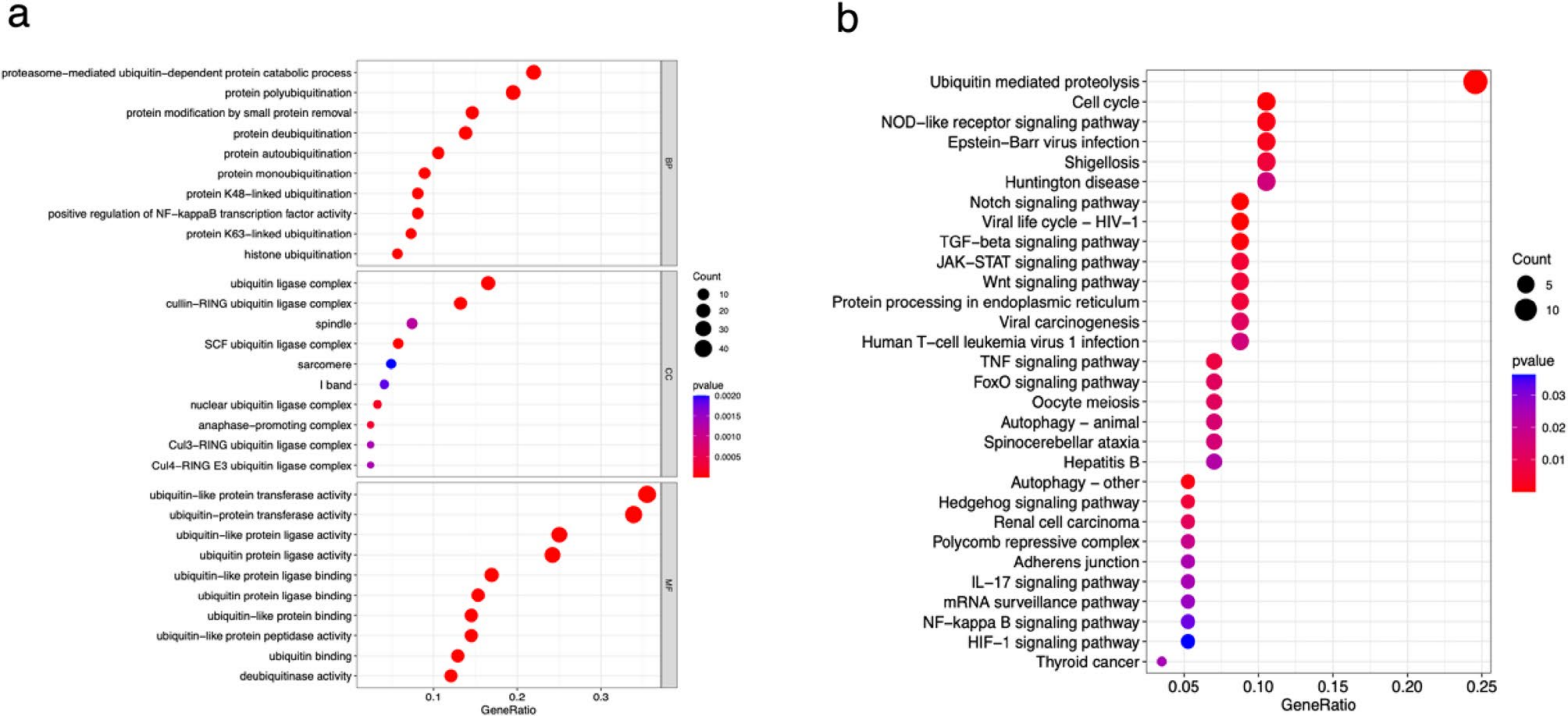
Bubble plots for GO (**a**) and KEGG (**b**) enrichment analyses. The horizontal axis represents the number of enriched genes, and the vertical axis displays the name of pathways.

Through the examination of GO and KEGG enrichment of the above 128 UbR-DEGs, we have identified the principal molecular functions and biological pathways of ubiquitination-related signatures to associate with AD. As depicted in Fig. 3a, the GO enrichment analysis of these UbR-DEGs revealed that the functions of BP primarily encompass proteasome-mediated ubiquitin-dependent protein catabolic process, protein polyubiquitination, protein modification by small protein removal, protein deubiquitination, and protein autoubiquitination, among others. The functions of CC were found to be closely linked to the ubiquitin ligase complex, cullin-RING ubiquitin ligase complex, spindle, SCF ubiquitin ligase complex, and sarcomere, among others. The functions of MF were predominantly enriched in ubiquitin-like protein or ubiquitin-protein transferase activity, ligase activity, and ligase binding, among others. In the KEGG enrichment analysis, the pathways identified by UbR-DEGs were primarily focused on Ubiquitin mediated proteolysis, Cell cycle, NOD-like receptor signaling pathway, Epstein–Barr virus infection, Shigellosis, Huntington disease, Notch signaling pathway, Viral life cycle-HIV-1, TGF-beta signaling pathway, and JAK-STAT signaling pathway, among others (Fig. 3b).

### Function and pathway enrichment in AD and normal groups

**Figure 4:**
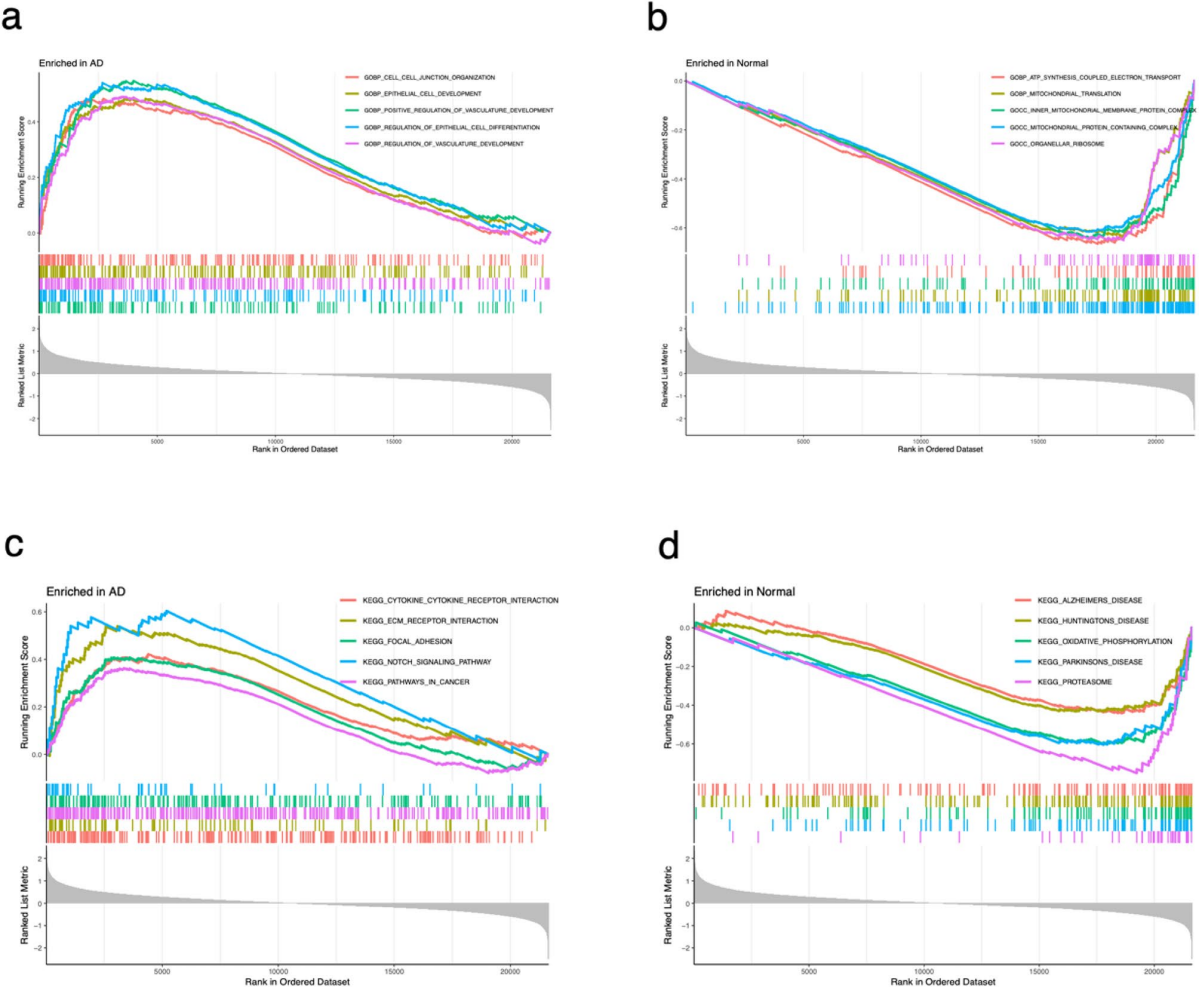
Plots of GSEA analysis. GO functional enrichment of AD (**a**) and normal group (**b**). KEGG pathway enrichment of AD (**c**) and normal group (**d**).

Through the utilization of GSEA analysis, we have gained insight into the enrichment of functions and pathways within both the AD and normal groups. Figure 4a illustrates the detection of significant enrichment in GO functions, specifically Cell–cell junction organization, Epithelial cell development, Positive regulation of vasculature development, Regulation of epithelial cell differentiation, and Regulation of vasculature development, within the AD group. Conversely, the normal group exhibited significant enrichment in Atp synthesis coupled electron transport, Mitochondrial translation, Inner mitochondrial membrane protein complex, Mitochondrial protein-containing complex, and Organellar ribosome, as depicted in Fig. 4b. Furthermore, Fig. 4c demonstrates that within the AD group, the KEGG pathway was significantly enriched in Cytokine cytokine receptor interaction, Ecm receptor interaction, Focal adhesion, Notch signaling pathway, and Pathways in cancer. On the other hand, the normal group exhibited significant enrichment in Alzheimer's disease, Huntingtons disease, Oxidative phosphorylation, Parkinsons disease, and Proteasome, as shown in Fig. 4d.

### Construction and validation of diagnostic models for AD based on six RUbR-DEGs

**Figure 5:**
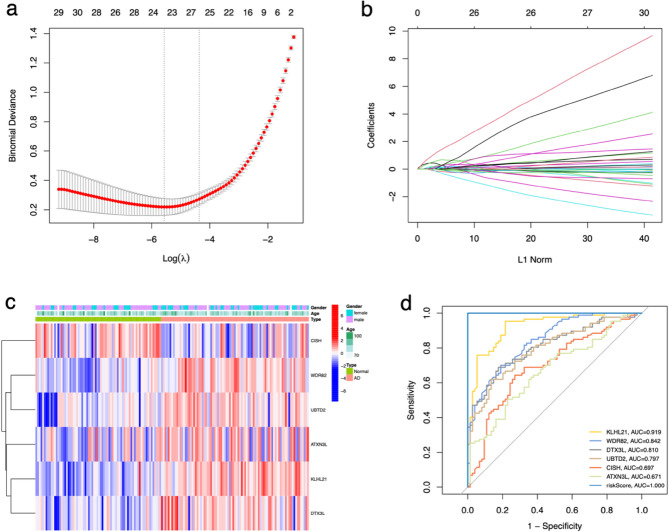
Screening of six RUbR-DEGs in AD. The deviance profile (**a**) and coefficient profile (**b**) of RUbR-DEGs screened by LASSO regression. (**c**) A heatmap of six RUbR-DEGs expressions between clinical phenotypes. Blue squares indicate deregulated genes; red squares indicate upregulated genes. (**d**) ROC curves of six RUbR-DEGs and Risk score. AUC is the area under the curve. Abscissa, 1-specificity (false positive rate). Ordinate, sensitivity (true positive rate).

The least absolute shrinkage and selection operator (LASSO) regression was used to avoid overfitting and screened 6 RUbR-DEGs (KLHL21, WDR82, DTX3L, UBTD2, CISH, and ATXN3L) (Fig. 5a,b). The differential expression of these RUbR-DEGs across various clinical phenotypes, such as age, gender, and type, was illustrated in Fig. 5c. Furthermore, we calculated the risk score for each sample using the risk calculation formula provided in the supplementary document (S1_Risk score.xls). In addition, the ROC curve demonstrated that all six RubR-DEGs exhibited an area under the ROC curve (AUC) exceeding 0.650. and the AUC value of the diagnostic model's parameter risk score equalled 1.000 (Table 2, Fig. 5d), suggesting that the constructed diagnostic model possessed exceptional precision and dependability in discerning patients with AD.

**Table 2:** Six RUbR-DEGs genes and their functions.

Gene name	Function	Impact AD or neurodegeneration
KLHL21	KLHL21 directly interacts with Aurora B and mediates the ubiquitination of Aurora B in vitro	Ubiquitin-dependent regulation of COPII coat size and function. Damage to CNS collagen is evident in disease and with aging and has major implications for driving neurodegeneration through its impact on inflammatory pathways in particular
WDR82	WDR82 is a negative regulator of virus-triggered type I IFNs pathway through mediating TRAF3 polyubiquitination status and stability on mitochondria	WDR82 mediates the polyubiquitination state and mitochondrial stability of TRAF3. The miR-590-5p inhibited the Traf3/MAPK P38 pathway, which means it plays an antiapoptotic role in AD
DTX3L	DTX3L is a multi-domain E3 ubiquitin ligase in which the N-terminus mediates protein oligomerisation, a middle D3 domain mediates the interaction with PARP9	DTX3L (Deltex E3 ubiquitin ligase 3 L) is an E3 ubiquitin ligase, a member of the deltex family. Stress response silencing by an E3 ligase mutated in neurodegeneration
UBTD2	UBTD2 is a ubiquitin (Ub) domain-containing protein first identified from dendritic cells and is implicated in the ubiquitination pathway	Six independent studies have shown that UBTD2 overlaps with abnormal DNA methylation genes in neurogenesis
CISH	CISH, participates within a multi-molecular E3 ubiquitin ligase complex, which ubiquitinates target proteins	Promote the E3 ubiquitin ligase to clear β-amyloid and hyperphosphorylated Tau by activating the PI3K/Akt signaling pathway in the hippocampus of AD mice, which is efficient in ameliorating pathological phenotypes
ATXN3L	ATXN3L is a deubiquitinating enzyme expressed in the brain	ATXN3L was among the genes in the X chromosome showing SNVs present in all the DNA samples from SAD patients. The gene is expressed in the brain and they have functions that could be related to SAD pathology

### The lncRNA-miRNA-mRNA (ceRNA) regulatory network based on six RUbR-DEGs in AD

**Figure 6:**
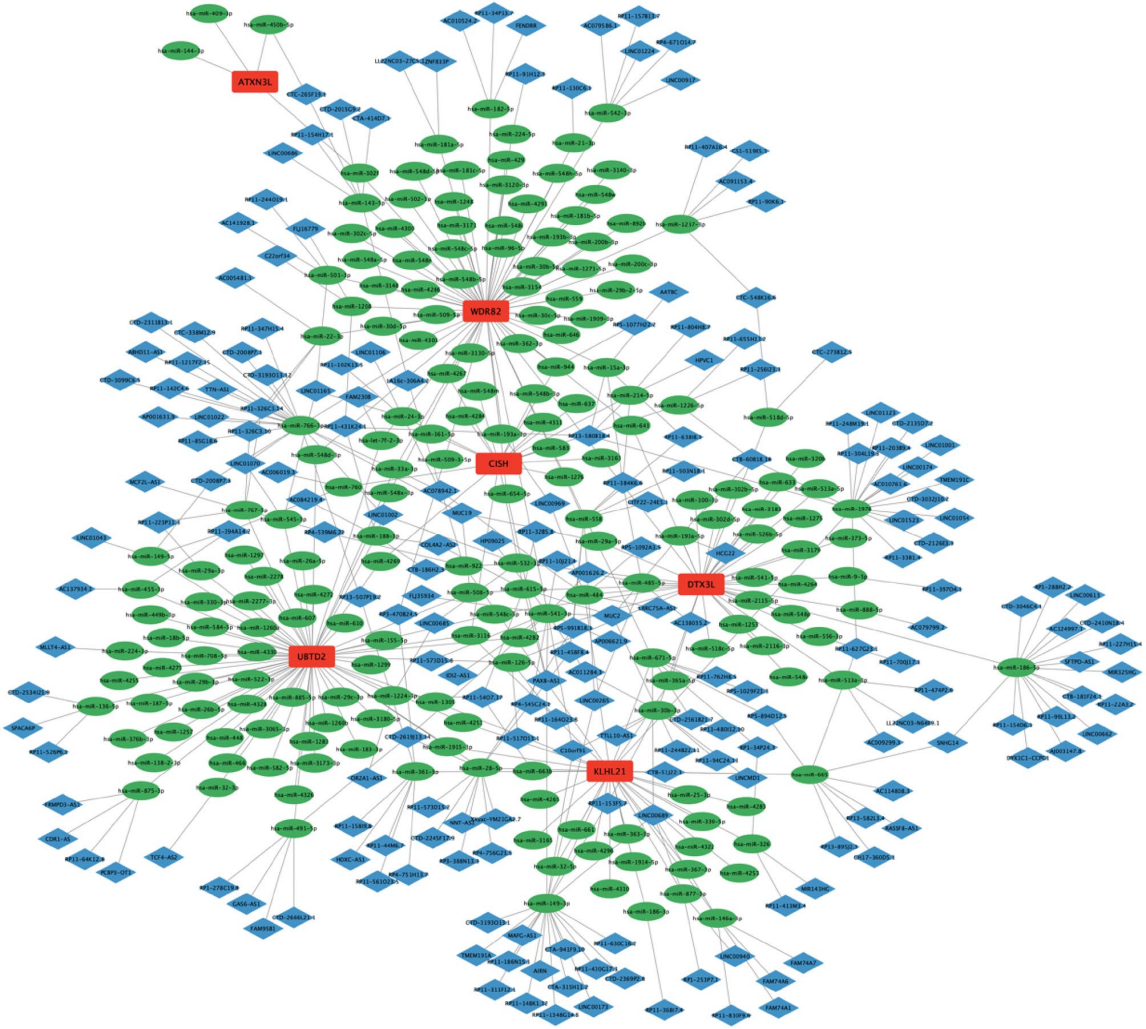
lncRNA-miRNA-mRNA (ceRNA) regulatory network. The red rectangular node represents mRNAs (RUbR-DEGs), the blue diamond node represents lncRNAs, the green oval node represents miRNAs, and the lines between them represent interaction pairs.

Using the databases TargetScan, miRanda and miRDB and custom Perl scripts, we obtained 221 mRNA-miRNA relationship pairs (see Supplementary document S2_mRNA-miRNA.xls). Then we determined 269 miRNA-lncRNA relationship pairs using the database spongeScan and Perl script (see Supplementary document S3_miRNA-lncRNA.xls). Finally, we used the software Cytoscape to construct the lncRNA-miRNA-mRNA (ceRNA) regulatory network (Fig. 6), which included 207 lncRNAs, 198 miRNAs, 6 mRNAs (RUbR-DEGs), and 411 interaction pairs.

## Discussion

AD is the most common type of elderly dementia, which can impair patients' thinking, memory, and independence, affecting their quality of life, and even leading to death^31,32^. The exact cause of AD has not yet been elucidated. Some studies have found that the typical histopathological changes of AD are amyloid protein deposition and neuronal fiber tangles in the brain^33^, and various theories are attempting to explain this change, including β-Amyloid protein waterfall theory, tau protein theory, neurovascular hypothesis, etc^34–36^. In the end, the nerve cells in the patient's brain "silently" atrophy or even die, or there are abnormalities in signal transmission between cells, leading to cognitive impairments such as memory, language, computation, and behavior^37^. Research has found that AD is the result of a combination of genes, lifestyle, and environmental factors, partially caused by specific genetic changes^4^. Ubiquitination of proteins is involved in the development of many diseases, including neurodegenerative diseases, such as AD^38,39^. Therefore, this study attempts to use comprehensive bioinformatics methods to elucidate the role of UbRGs in AD and their specific molecular functions and construct a risk diagnosis model for AD based on RUbR-DEGs.

In this study, we first collected 161 samples from the GEO database (including 87 in the AD group and 74 in the normal group). Subsequently, through differential expression analysis and the iUUCD 2.0 database, we obtained 3450 DEGs and 806 UbRGs. After taking the intersection, we obtained 128 UbR-DEGs. This indicated that these UbR DEGs play a key role in the occurrence and development of AD.

Secondly, by conducting GO and KEGG enrichment analysis on these 128 UbR-DEGs, we identified the main molecular functions and biological pathways related to AD. The GO enrichment analysis revealed that the functions are found to be closely linked to protein polyubiquitination, modification, deubiquitination and autoubiquitination, the ubiquitin ligase complex, spindle, sarcomere, ubiquitin-like protein or ubiquitin-protein transferase activity, ligase activity, and ligase binding, among others. In the KEGG enrichment analysis, the pathways identified by UbR-DEGs were primarily focused on Ubiquitin mediated proteolysis, Cell cycle, NOD-like receptor signaling pathway, Notch signaling pathway, TGF-beta signaling pathway, and JAK-STAT signaling pathway, among others. Furthermore, through the utilization of GSEA analysis, we have gained insight into the enrichment of functions and pathways within both the AD and normal groups. Among them, GO functions were mainly enriched in Cell–cell junction organization, Epithelial cell development, Positive regulation of vasculature development, Regulation of epithelial cell differentiation, and Regulation of vasculature development, within the AD group. And KEGG pathways were significantly enriched in Cytokine cytokine receptor interaction, Ecm receptor interaction, Focal adhesion, Notch signaling pathway, and Pathways in cancer.

Further, using lasso regression analysis and cross-validation techniques, we identified 22 characteristic genes associated with AD. Subsequently, we constructed a logistic regression model and optimized it, resulting in the identification of 6 RUbR-DEGs: *KLHL21, WDR82, DTX3L, UBTD2, CISH,* and *ATXN3L.* A Gómez Ramos et al.^40^ sequenced the Exon group of brain samples from sporadic AD (SAD) patients and normal controls. They found more single nucleotide variations (SNV) in the genes on the X chromosome of SAD patients, and verified these variants through Sanger sequencing. Two new gene variants were found in the loci related to the ubiquitination (*UBE2NL* and *ATXN3L*). UBE2NL is another protein related to the ubiquitination-de-ubiquitination process. It is also expressed in brain and it participates in parkin-dependent mitophagy, a process that can be dysregulated in AD. ATXN3L is a deubiquitinating enzyme expressed in brain and associated with Machado-Joseph disease. ATXN3L and UBE2NL were among the genes in the X chromosome showing SNVs present in all the DNA samples from SAD patients. These two genes are expressed in the brain and they have functions that could be related to SAD pathology. We compared the present data with previous loci detected in GWAS studies., We found that a SNV at ATXN3L, locus chrx: 13337059, has been already reported (http://www.gwascentral.org/). It has been found that ubiquitin carboxyl-terminal Hydrolase L1 (*UCH-L1*) is a kind of deubiquitinating enzyme, which plays a regulatory role in proteins that target Proteasome degradation^41^. *UCH-L1* is highly expressed in neurons and has been shown to promote cell viability and maintain neuronal integrity. The expression of UCH-L1 can salvage synaptic dysfunction and memory deficits in AD model mice^42,43^. Wang et al.^44^ found that the inflammatory stimulator lipopolysaccharide and TNF-α activate NF-κB signaling by inhibiting its transcription leading to decreased *UCH-L1* gene expression, suggesting that inflammation may impair the normal function of neurons through the interaction of NF-κB and *UCH-L1*. The loss of UCH-L1 activity coupled with the gain of proteinopathy function are linked to neurodegeneration such as Parkinsonism and Alzheimer's disease. In addition, the ROC result showed that the diagnostic model we built has excellent accuracy and reliability in identifying AD patients.

Finally, we constructed a lncRNA-miRNA-mRNA (ceRNA) regulatory network for AD based on six RUbR-DEGs, further elucidating the interaction between UbRGs and lncRNA, miRNA. This has important reference significance for us to deeply understand the molecular pathogenic mechanism of AD and develop potential drug targets.

However, this study also has certain limitations that need to be addressed in future research. For example, due to the limitations of database samples, it is mainly based on ubiquitination-related genes, lacking additional information on other genes involved in AD, molecular pathways, and how they are more generally related to ubiquitin, and lacking additional integrated datasets for extensive gene ontology analysis. In addition, there is a lack of subgroup analysis of AD pathological subtypes and more detailed clinical parameters to improve the construction of risk diagnosis models. At the same time, more samples need to be collected to confirm our findings through wet experiments.

### Supplementary Information

Supplementary Information 1.

Supplementary Information 2.

Supplementary Information 3.

## Data Availability

The dataset used and/or analyzed during this study may be granted by contacting the corresponding author.

## References

[1] Huang N, Huang J, Feng F, Ba Z, Li Y, Luo Y (2022). Tanshinone ΙΙA-incubated mesenchymal stem cells inhibit lipopolysaccharide-induced inflammation of N9 cells through TREM2 signaling pathway. Stem Cells Int..

[2] Xu X, Ruan W, Liu F, Gai Y, Liu Q, Su Y (2021). 18F-APN-1607 tau positron emission tomography imaging for evaluating disease progression in Alzheimer's disease. Front. Aging Neurosci..

[3] Liu X-Y, Yang L-P, Zhao L (2020). Stem cell therapy for Alzheimer's disease. World J. Stem Cells.

[4] Suh Y, Ah Y-M, Han E, Jun K, Hwang S, Choi KH (2020). Dose response relationship of cumulative anticholinergic exposure with incident dementia: Validation study of Korean anticholinergic burden scale. BMC Geriatr..

[5] Meguro K, Akanuma K, Meguro M, Kasai M, Ishii H, Yamaguchi S (2015). Lifetime expectancy and quality-adjusted life-year in Alzheimer's disease with and without cerebrovascular disease: Effects of nursing home replacement and donepezil administration—a retrospective analysis in the Tajiri Project. BMC Neurol..

[6] Nozawa K, Fujihara Y, Devlin DJ, Deras RE, Kent K, Larina IV (2022). The testis-specific E3 ubiquitin ligase RNF133 is required for fecundity in mice. BMC Biol..

[7] Liao Y, Sumara I, Pangou E (2022). Non-proteolytic ubiquitylation in cellular signaling and human disease. Commun. Biol..

[8] Fu L, Lu K, Jiao Q, Chen X, Jia F (2023). The regulation and double-edged roles of the deubiquitinase OTUD5. Cells.

[9] Huang Z, Yang P, Ge H, Yang C, Cai Y, Chen Z (2020). RING finger protein 38 mediates LIM domain binding 1 degradation and regulates cell growth in colorectal cancer. Onco Targets Ther..

[10] Lu X, Yang B, Qi R, Xie Q, Li T, Yang J (2023). Targeting WWP1 ameliorates cardiac ischemic injury by suppressing KLF15-ubiquitination mediated myocardial inflammation. Theranostics.

[11] Geiszler PC, Ugun-Klusek A, Lawler K, Pardon M-C, Yuchun D, Bai L (2018). Dynamic metabolic patterns tracking neurodegeneration and gliosis following 26S proteasome dysfunction in mouse forebrain neurons. Sci. Rep..

[12] Xiang C, Wu J, Yu L (2022). Construction of three-gene-based prognostic signature and analysis of immune cells infiltration in children and young adults with B-acute lymphoblastic leukemia. Mol. Genet. Genomic Med..

[13] Zhang Q, Yang P, Pang X, Guo W, Sun Y, Wei Y, Pang C (2022). Preliminary exploration of the co-regulation of Alzheimer's disease pathogenic genes by microRNAs and transcription factors. Front. Aging Neurosci..

[14] Yu Z-H, Feng S-T, Zhang D, Cao X-C, Yu Y, Wang X (2021). The functions and prognostic values of m6A RNA methylation regulators in thyroid carcinoma. Cancer Cell Int..

[15] Bao Y, Wang L, Yu F, Yang J, Huang D (2023). Parkinson's disease gene biomarkers screened by the LASSO and SVM algorithms. Brain Sci..

[16] Yang S, Yao B, Wu L, Liu Y, Liu K, Xu P (2021). Ubiquitin-related molecular classification and risk stratification of hepatocellular carcinoma. Mol. Ther. Oncolytics.

[17] Pan Y, Wu L, He S, Wu J, Wang T, Zang H (2022). Identification of hub genes and immune cell infiltration characteristics in chronic rhinosinusitis with nasal polyps: Bioinformatics analysis and experimental validation. Front. Mol. Biosci..

[18] Farooq U, Wang H, Hu J, Li G, Jehan S, Shi J (2023). Polydatin inhibits hepatocellular carcinoma cell proliferation and sensitizes doxorubicin and cisplatin through targeting cell mitotic machinery. Cells.

[19] Ma P, Yue L, Zhang S, Hao D, Wu Z, Xu L (2020). Target RNA modification for epigenetic drug repositioning in neuroblastoma: Computational omics proximity between repurposing drug and disease. Aging.

[20] Zhang Y, Jiang W, Xia Q, Lin J, Xu J, Zhang S (2022). Construction of a potential microRNA and messenger RNA regulatory network of acute lung injury in mice. Sci. Rep..

[21] Bi Y-H, Wang J, Guo Z-J, Jia K-N (2022). Characterization of ferroptosis-related molecular subtypes with immune infiltrations in neuropathic pain. J. Pain Res..

[22] Liu J, Sun T, Liu S, Liu J, Fang S, Tan S (2022). Dissecting the molecular mechanism of cepharanthine against COVID-19, based on a network pharmacology strategy combined with RNA-sequencing analysis, molecular docking, and molecular dynamics simulation. Comput. Biol. Med..

[23] Yu Y, Zeng Y, Xia X, Zhou J-G, Cao F (2021). Establishment and validation of a prognostic immune signature in neuroblastoma. Cancer Control.

[24] Cai J, Li H, Zhang C, Chen Z, Liu H, Lei F (2021). The neutrophil-to-lymphocyte ratio determines clinical efficacy of corticosteroid therapy in patients with COVID-19. Cell Metab..

[25] Wang H, Zhang Y, Zheng C, Yang S, Chen X, Wang H, Gao S (2022). A 3-gene-based diagnostic signature in Alzheimer's disease. Eur. Neurol..

[26] Stefani S, Kousiappa I, Nicolaou N, Papathanasiou ES, Oulas A, Fanis P (2020). Neurophysiological and genetic findings in patients with juvenile myoclonic epilepsy. Front. Integr. Neurosci..

[27] Yang S, Liu T, Cheng Y, Bai Y, Liang G (2019). Immune cell infiltration as a biomarker for the diagnosis and prognosis of digestive system cancer. Cancer Sci..

[28] Zhao B, Yu Q, Li H, Guo X, He X (2014). Characterization of microRNA expression profiles in patients with intervertebral disc degeneration. Int. J. Mol. Med..

[29] Xu Y, Zou X, Mei J (2022). The risk correlation between N7-methylguanosine modification-related lncRNAs and survival prognosis of oral squamous cell carcinoma based on comprehensive bioinformatics analysis. Appl. Bionics Biomech..

[30] Huang X, Liufu Q, Xu R, Chen X, Liu M, Han J (2022). Integrating lncRNAs and mRNAs expression profiles in penicillin-induced persistent chlamydial infection in HeLa cells. Front. Mol. Biosci..

[31] Mantoani SP, Chierrito TPC, Vilela AFL, Cardoso CL, Martínez A, Carvalho I (2016). Novel triazole-quinoline derivatives as selective dual binding site acetylcholinesterase inhibitors. Molecules.

[32] Cantarella G, Di Benedetto G, Puzzo D, Privitera L, Loreto C, Saccone S (2015). Neutralization of TNFSF10 ameliorates functional outcome in a murine model of Alzheimer's disease. Brain.

[33] He X-F, Xu J-H, Li G, Li M-Y, Li L-L, Pei Z (2020). NLRP3-dependent microglial training impaired the clearance of amyloid-beta and aggravated the cognitive decline in Alzheimer's disease. Cell Death Dis..

[34] Wu Q, Naeem A, Zou J, Yu C, Wang Y, Chen J (2022). Isolation of phenolic compounds from raspberry based on molecular imprinting techniques and investigation of their anti-Alzheimer's disease properties. Molecules.

[35] He K-C, Chen Y-R, Liang C-T, Huang S-J, Tzeng C-Y, Chang C-F (2020). Conformational characterization of native and L17A/F19A-substituted Dutch-type β-amyloid peptides. Int. J. Mol. Sci..

[36] Shams S, Granberg T, Martola J, Charidimou A, Li X, Shams M (2017). Cerebral microbleeds topography and cerebrospinal fluid biomarkers in cognitive impairment. J. Cereb. Blood Flow Metab..

[37] Qin R, Zhou D, Wang J, Hu H, Yang Y, Yao X (2012). Compound Danshen tablets downregulate amyloid protein precursor mRNA expression in a transgenic cell model of Alzheimer's disease: Effects and a comparison with donepezil. Neural Regen. Res..

[38] Lu M, Chen W, Zhuang W, Zhan X (2020). Label-free quantitative identification of abnormally ubiquitinated proteins as useful biomarkers for human lung squamous cell carcinomas. EPMA J..

[39] Li Y, Xie P, Lu L, Wang J, Diao L, Liu Z (2017). An integrated bioinformatics platform for investigating the human E3 ubiquitin ligase-substrate interaction network. Nat. Commun..

[40] Gómez-Ramos A, Podlesniy P, Soriano E, Avila J (2015). Distinct X-chromosome SNVs from some sporadic AD samples. Sci. Rep..

[41] Zhang H, Luo W, Sun Y, Qiao Y, Zhang L, Zhao Z (2016). Wnt/β-catenin signaling mediated-UCH-L1 expression in podocytes of diabetic nephropathy. Int. J. Mol. Sci..

[42] Liu MC, Akinyi L, Scharf D, Mo J, Larner SF, Muller U (2010). Ubiquitin C-terminal hydrolase-L1 as a biomarker for ischemic and traumatic brain injury in rats. Eur. J. Neurosci..

[43] Gong B, Radulovic M, Figueiredo-Pereira ME, Cardozo C (2016). The ubiquitin-proteasome system: Potential therapeutic targets for Alzheimer's disease and spinal cord injury. Front. Mol. Neurosci..

[44] Wang R, Zhang M, Zhou W, Ly PTT, Cai F, Song W (2011). NF-κB signaling inhibits ubiquitin carboxyl-terminal hydrolase L1 gene expression. J. Neurochem..

